# A study of bacteria producing carbonic anhydrase enzyme for CaCO_3_ precipitation and soil biocementation

**DOI:** 10.1007/s11356-024-34077-0

**Published:** 2024-07-08

**Authors:** Wilson Mwandira, Maria Mavroulidou, Martijn Timmermans, Michael John Gunn, Christopher Gray, Leonardo Pantoja-Muñoz, Diane Purchase

**Affiliations:** 1 Division of CIBSE, School of the Built Environment and Architecture, London South Bank University, London, UK; 2 Department of Natural Sciences, Faculty of Science and Technology, Middlesex University, London, UK

**Keywords:** Carbonic anhydrase enzyme, Microbiological study, Calcite precipitation, Biocementation, Ground improvement

## Abstract

We study the carbonic anhydrase (CA) pathway using autochthonous CA-producing bacteria as a means of inducing calcite precipitation, which acts as a biocement to improve the engineering soil properties. Forty different microbial strains producing CA were isolated from the foundation soil of a railway embankment in Prickwillow, UK. Three of the best CA-producing strains were selected and identified by DNA sequencing as *Bacillus licheniformis*, *Bacillus toyonensis* and *Bacillus pumilus* with CA activity values respectively of 1.79 U/ml, 1.42 U/ml and 1.55 U/ml*.* To optimise the treatments, we investigated the effect of pH, temperature, zinc co-factor and cementation solution molarity on the growth and CA activity and bioprecipitates, with CO_2_ added in the form of bicarbonate. Scanning electron microscope (SEM) analysis of the bioprecipitates showed that these had characteristic morphologies of calcite and vaterite crystals. The formation of calcite was further corroborated by FT-IR and Raman analysis of bioprecipitates. The precultured bacteria were injected into the fine-grained soil together with cementation solution. Unconfined compressive strength in treated soil increased up to 1 MPa, and its calcium carbonate content increased by 2.78%. This, as well as the stability of the treated soil upon water immersion, proved the biocementation of the fine-grained soil. These findings suggest the potential of employing the CA biocementation route for soil stabilisation pending further development of the technique.

**Supplementary Information:**

The online version contains supplementary material available at 10.1007/s11356-024-34077-0.

## Introduction

Population shifts from rural to urban areas are forcing engineers to construct on inferior ground. To enable this, ground improvement has increasingly been used in geotechnical engineering practice (Astaraki and Esmaeili 2022; Safdar et al. 2022). Different ground improvement methods include mechanical, physical and chemical techniques; these methods suffer from several shortcomings linked to costs, heavy machinery and disturbance of services and environmental or human health side effects (Naeimi and Haddad 2020). Chemical ground improvement methods in particular use most commonly lime (Kichou et al. 2022, Zhang et al. 2014 and 2015) and Portland cement (PC) (Lorenzo and Bergado 2006), whose production consumes a high amount of energy and emits 8–10% of global CO_2_ (Mavroulidou et al. 2022a). Efforts are being made to develop alternative forms of cement for ground improvement, which are ecologically friendlier and require less energy in their manufacture. Examples of such cements include alkali activated/geopolymer cements (e.g. Singhi et al 2017; Coudert et al. 2019; Rios et al 2019; Banaś et al 2020; Mavroulidou et al. 2022b; Mavroulidou et al. 2023) and biocements (e.g. Whiffin 2004; van Paassen et al. 2010; Gomez et al 2017; Chittoori et al. 2018; Mwandira et al. 2019; Mwandira et al. 2021; Lee et al. 2023). Biocements have emerged as a promising environmentally friendly alternative to PC as they are biomimetic (nature-based). Biocements harness the metabolic action of microorganisms to precipitate biominerals acting as binders of soil particles, which increase soil stiffness and strength. The most commonly precipitated minerals are carbonates, mainly calcium carbonate. The acronym MICP (microbially induced carbonate precipitation) is used to refer to this process, although precipitation of different biominerals (other than carbonates) is possible and has also been studied (e.g. Amarakoon et al. 2014).

Different bacterial species precipitate carbonates in their natural habitats via various metabolic pathways that include ureolysis, denitrification, methane oxidation, photosynthesis, sulfate reduction, iron reduction and the interconversion between CO_2_ and the bicarbonate ion HCO_3_^–^ mediated by heterotrophic carbonic anhydrase (CA)–producing bacteria (Mwandira et al 2023b). Of these, the CA pathway is of particular interest, as it can produce biominerals for biocementation via the consumption of CO_2_ according to the following chemical reactions: first, CA utilises CO_2_, to form hydrated aqueous CO_2_ (aq) according to Eq. (1); aqueous CO_2_ reacts with H_2_O and forms H_2_CO_3_ according to Eq. (2). The H_2_CO_3_ dissociation products ionise and form CO_3_^2−^ and H_2_O according to Eqs. (3 and 4). Ca^2+^ ions then need to be supplied to react with CO_3_^2−^ (Eq. 5) to form CaCO_3_ (Mwandira et al. 2023c):

1 $${CO}_{2}\left(g\right)\leftrightarrow{CO}_{2}\left(aq\right)$$

2 $${CO}_{2}\left(aq\right)+{H}_{2}O\leftrightarrow {H}_{2}{CO}_{3}$$

3 $${H}_{2}{CO}_{3}\leftrightarrow {H}^{+}+{{HCO}_{3}}^{-}$$

4 $$2H{{CO}_{3}}^{-}+2{OH}^{-}\leftrightarrow 2{{CO}_{3}}^{2-}+2{H}_{2}O$$

5 $${Ca}^{2+}+{{CO}_{3}}^{2-}\leftrightarrow Ca{CO}_{3}\downarrow$$

Based on life cycle assessment studies, the carbonic anhydrase metabolic pathway was found to be the most sustainable pathway in comparison to other biocementation pathways, in terms of CaCO_3_ production (Porter et al 2021). There are, however, very limited studies on CA biocementation for ground improvement with inconclusive results (see the literature review by Mwandira et al 2023b).

To address this knowledge gap, this research builds on the promising results of work presented at the CEMEPE10 conference by Mwandira et al. (2023d) studying the ability of CA-producing bacteria in precipitating calcite and biocementing soil, by adding CO_2_ in the form of bicarbonate. This can be relevant to the method of capturing industrial CO_2_ in the form of bicarbonate (Vaughan 2022). The particular novelties of this research are (a) the isolation of CA producing bacteria which are naturally occurring in the soil to be biocemented and the in-depth microbiological study and molecular characterisation of the isolates and (b) the proof of the optimised treatments presented in this study, towards fine-grained soil biocementation (other researchers only studied sand soil CA biocementation). By proving soil biocementation using the CA pathway, this research contributes to developing an eco-friendly biocementation technique with the advantage of stabilising the soil, which is used as a carbon sink for captured waste CO_2_ during the stabilisation process. The research thus actively aligns with the extensive efforts for climate change mitigation and adaptation as it allows for simultaneously decarbonising the ground engineering industry and enhancing the resilience of civil infrastructure.

## Materials and methods

### Soil

**Fig. 1:**
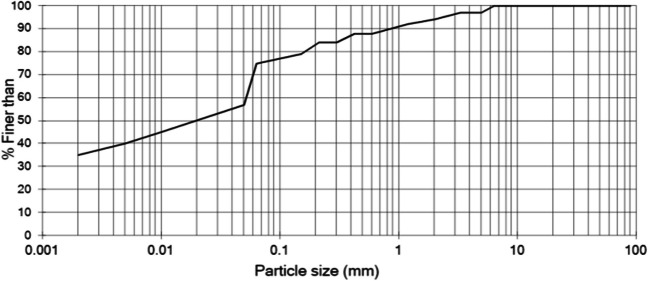
PSD of soil

**Fig. 2:**
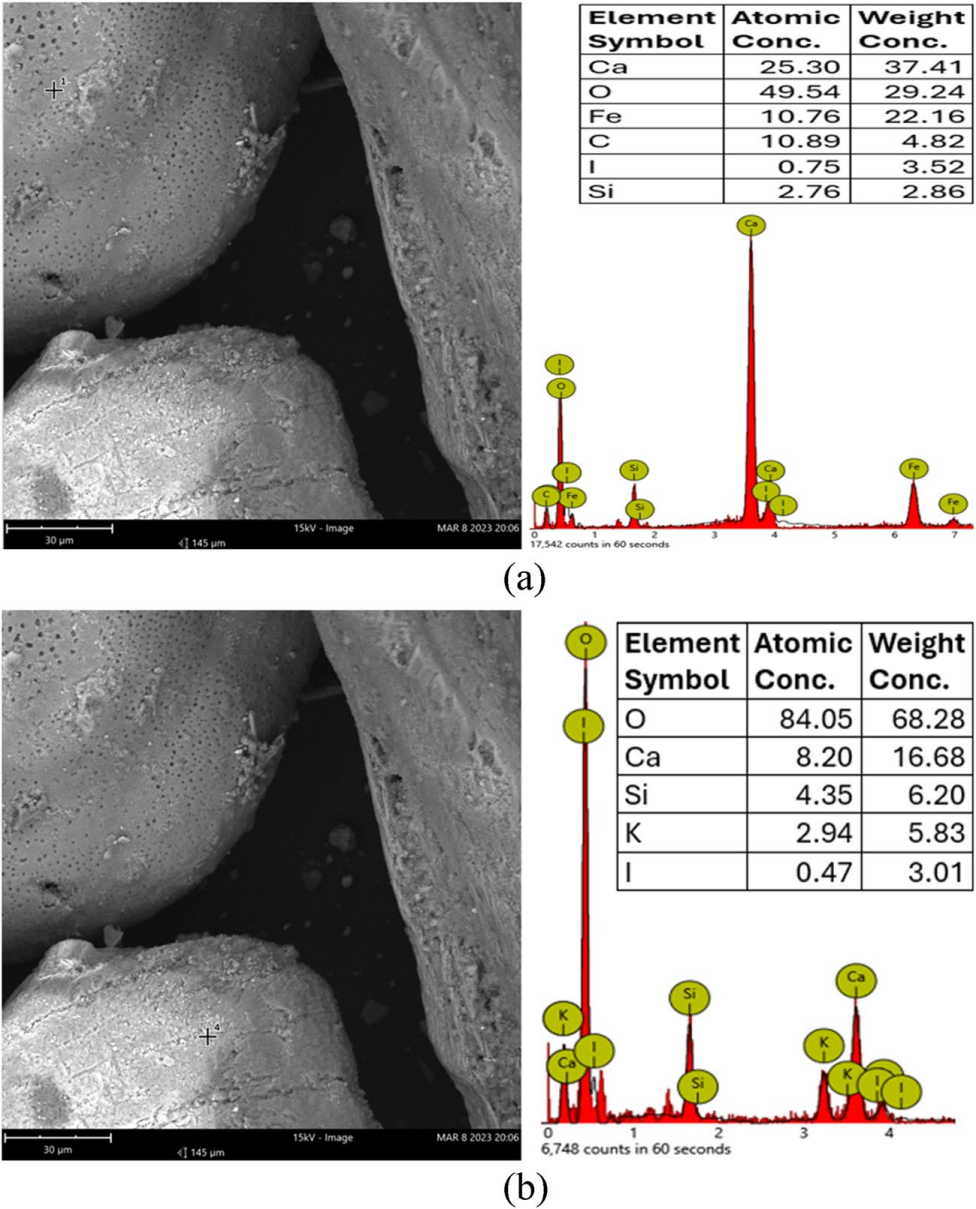
**a**, **b** Material analysis of the untreated soil based on SEM and EDS indicatively shown on two different sites of the soil sample (see photos **a** and **b** respectively)

The sample originated from 0.6 to 1.1-m depth above the groundwater table; it was extracted from a trial pit next to a railway embankment in Prickwillow, UK (the same geo-location as in Mwandira et al 2023a but a different depth). The soil from this layer was fine-grained but not plastic, unlike deeper soil layers of the same site (studied e.g. in Mwandira et al 2023a). Its particle size distribution (PSD) based on sieving and then hydrometer testing (BSI 2022a) is shown in Fig. 1. Material characterisation of the soil based on SEM–EDS analysis is shown in Fig. 2.

From duplicate samples, the soil was found to have a pH of 7.15 (suspended in distilled water and tested according to BSI, 2005), a natural gravimetric moisture content of 32.4% (tested according to BSI, 2022b), an organic content of 7% (tested according to ASTM 2014) and a natural CaCO_3_ content of 4.77% (tested according to ASTM 2021). Based on these characteristics, our first assessment was that although the soil did not have a high organic content (unlike other foundation soil layers in the area, e.g. in Safdar et al 2021a,b & 2022) which can be potentially challenging for biocementation, the fine-grained size of the soil still poses potential challenges for biocementation treatment compared to sands (sands constitute the predominant soil type in the majority of other research and field applications of biocementation). The low hydraulic conductivity of fine-grained soils renders the flow and transport of the treatment solutions difficult, and for bioaugmentation, the precultured bacteria implemented into the soil must be able to pass through the narrow pore throats of the fine-grained soil, which would need to be bigger than the size of the bacteria.

### Microbiological study

#### Isolation and characterization of CA-producing microbes

The microbes found to produce CA were isolated from 1 g of soil placed in 10-ml deionized, sterile water and homogenized using a vortex mixer. The soil suspension was serially diluted to 10^−4^ using sterile deionised water, and 0.1 ml of the dilution was plated out on peptone agar medium containing 3 mM of *p*-nitrophenyl acetate (referred to as p-NPA), an indicator of CA-positive microbes. Colonies able to produce CA generated an intense yellow colour as p-NPA hydrolysed into para-nitrophenol (pNP). The plates were put in an incubator at 30 °C for 72 h. After incubation, the soil yielded 40 CA-producing bacteria with different morphologies. An initial identification of microorganisms was performed using MALDI-TOF/TOF MS proteomic-based biotyping with a Bruker Daltonics MALDI Biotyper as described in Mwandira et al (2023a).

#### Bacterial growth, CA enzyme production and characterization

Of the 40 isolates showing CA activity, a preliminary screening based on the intensity of the yellow colour was carried out. The best three strains were selected for further study and quantification of their enzymatic activities. These three strains were preliminarily labelled as strain U-1, strain U-21 and strain U-26 until further identification through DNA sequencing, as described below. Pure single colonies of the three strains were aseptically inoculated in nutrient broth (50 ml) and incubated at 37 °C for 24 h. A 37 °C temperature was chosen based on the temperature identified for optimal CA enzyme activity elsewhere in the literature (Mwandira et al. 2023d). We determined CA colourimetrically (see Martin et al. 2009). Namely, the activity for *p*-nitrophenyl acetate hydrolysis was assessed using 1.35 ml of reaction mixture with 3 mM *p*-nitrophenyl acetate in phosphate buffer (0.13 M and pH 7.2) at room temperature. The reaction continued for 5 min, and the optical density (OD) change at 348 nm was measured using UV–Vis spectrophotometry. The quantity of *p*-nitrophenol generated per unit of time was used to characterise the CA enzyme activity as in Nathan and Ammini (2019):

6 $$CA\;activity \left(\frac{\mathrm{U}}{\mathrm{ml}}\right)=\frac{(\Delta {A}_{348}T-\Delta {A}_{348}B)\times 1000}{5 \times Volume}$$

where $\Delta {A}_{348}T$ is the final absorbance reading; $\Delta {A}_{348}B$ is the initial uncatalyzed reaction at a 348-nm wavelength. Volume is the volume of bacterial suspension added to the cell. 1 U (μmol/min) is the amount of the enzyme catalysing the conversion of 1 mM of substrate per min.

To determine the intracellular versus extracellular activity, the following method was used: 10 ml of bacteria culture was placed in a centrifuge tube (1) and centrifuged in 8000 rpm for 5 min. Following this, pellet cells were lodged at the bottom of the tube (tube 1) whilst the solution remaining in the tube (supernatant) was transferred to another centrifuge tube (tube 2). PBS buffer was then added to the pellet cells in tube (1), and it was mixed and shaken. Tube (1) was then sonicated at a 37 Hz frequency for 5 min; it was then again centrifuged at 8000 rpm for 5 min. The enzyme was then measured in both tube (1) containing the cells and tube (2) containing the supernatant to determine the intracellular and extracellular enzyme, respectively, following the method used for finding the CA enzyme using a spectrophotometer as described earlier.

#### Optimisation of CA activity

To optimise CA activity, we performed an investigation of the effect pH, temperature, zinc cofactor and the molarity of the cementation solution (consisting of calcium acetate and sodium bicarbonate) on the growth of the bacteria and enzymatic activity. Bacterial growth was determined using UV–Vis spectrophotometry that recorded OD readings at 600 nm for 96 h. Following sterile inoculation, we cultured the medium at a constant temperature oscillation incubator (120 rpm) at 5 ℃, 15 ℃, 25 ℃, 30 ℃ and 37 ℃ respectively for 24 h before measuring the OD_600_ value and CA in the medium solution. Similarly, the effect of the pH was measured for a range of pH = 6–11 by adjusting the pH using NaOH solution and measuring the OD_600_ and CA in the solution as described previously. Finally, the effect of zinc acting as a metallocofactor for CA was evaluated by adding Zn to the assay reaction mix at concentrations of 0, 1, 5, 10 and 20 mM respectively. The enzyme activity was then determined according to Eq. (6) for different Zn concentrations. The tests were performed in triplicate.

An additional investigation examined the effect of the source of calcium (i.e. calcium salt supplied) on bacterial growth and enzymatic activity. This investigation was carried out indicatively for one of the selected strains, namely U-1.

#### DNA sequencing and analysis

The three selected strains (labelled respectively as strain U-1, strain U-21 and strain U-26) were prepared for sequencing. For each of the three strains, a 1-ml overnight culture was pelleted and resuspended in 600-µl 50 mM EDTA containing 120-mg lysozyme (Sigma-Aldrich, St. Louis, USA). DNA was then extracted using a Promega Wizard Genomic DNA purification kit using the manufacturer’s protocol for Gram-positive bacteria. We assessed the extracted DNA quality and quantity using a NanoDrop spectrophotometer (supplied by ThermoFisher Scientific) and a Genomic DNA ScreenTape essay on a TapeStation 4200 System (Agilent, Santa Clara, USA). The DNA samples were shipped in icepacks to Novogene (Cambridge, UK) for library preparation and sequencing. In brief, before Illumina adapters were ligated, the DNA was fragmented, end repaired and A-tailed. The ligated fragments were size-selected, PCR amplified and purified to obtain the final libraries. The libraries (insert + adapters length: 320–520 bp) were sequenced on an Illumina NovaSeq 6000 (paired-end; 2*150 bp).

We processed raw reads using Trimmomatic v0.39 (Bolger et al 2014), removing remaining adapters and any 5′ and 3′ bases with a quality value below 20. IDBA was used to assemble the remaining sequence reads (Peng et al 2012) with ‘*mink*’ set to 80 and ‘*maxk*’ set to 124, but otherwise default settings. The genomes were annotated using pgap (Tatusova et al 2016; quality and contamination scores of the three genome assemblies were determined using Quast (Gurevich et al 2013) and CheckM (Parks et al 2015)), respectively. The assembled genomes were combined with chromosome-level assembled, full-length *Bacillaceae* genomes (downloaded from the NCBI Datasets database on 02 Nov. 2023) for phylogenetic reconstruction using the GToTree (Lee 2019) pipeline, allowing for steps to be run in parallel (Tange 2018). For this, 74 single-copy orthologues were targeted using HMMER3 (Eddy 2011) and the provided ‘Bacteria’ HMM file. Before concatenating, the gene sequences were translated using Prodigal v2.6.3, aligned using Muscle 5.1 (Edgar 2004), trimmed using TrimAl v1.4 (Capella-Gutiérrez et al 2009). Then, phylogenetic analyses were performed using FastTree Version 2.1.11(see Price et al. 2010) (a JTT model of protein evolution, CAT rate approximation with 20 rate categories, and Shimodaira-Hasegawa test with 1000 bootstrap replicates to calculate support values). TaxonKit (Shen and Xiong 2019) was used to retrieve and add NCBI taxonomy info to the taxon labels. The tree was visualised and formatted using FigTree v1.4.4 (Rambaut 2018). Putative carbonic anhydrase genes were pinpointed by searching the pgap annotations.

#### CaCO_3_ production in aqueous solution

Bioprecipitation due to the selected isolates was examined using equimolar solutions of sodium bicarbonate and calcium acetate (0.1, 0.25, 0.5, 0.75 and 1.0 M respectively). Note that for the main investigations, we used sodium bicarbonate to obtain the required CO_2_ instead of CO_2_ directly injected to the soil. The bacterial isolates were precultured for 24 h in 10-ml liquid nutrient medium. The nutrient medium composition is shown in Table 1. We incubated aerobically inoculant solutions at 37 °C for 48 h with 120 rpm shaking; we then inoculated 1 ml of preculture into broth (100 ml) and grew the inoculum at 37 °C for 96 h with continuous aeration at 120 rpm. The overnight microbial culture was diluted to 0.5 MacFarland standard to reach a cell concentration of 9 × 10^8^ cells/l at OD_600_; this concentration was subsequently used in the bioaugmentation treatments using cementation solutions with the molarities mentioned above. The mixes were incubated at 30 °C for 6 h and shaken at 160 rpm; subsequently, to collect the precipitate, they were centrifuged at 15,000 rpm for 5 min. An additional qualitative investigation to attest the possibility of obtaining bioprecipitation due to atmospheric CO_2_ capturing (without the use of bicarbonate) in 0.1 M calcium acetate solution was also performed in triplicate. Namely, the substrates were bioaugmented using the same cell concentration of 9 × 10^8^ cells/l (one strain at a time) and following the same inoculation procedure as described earlier, however without attempting to control the ingress of other microorganisms in order to mimic the conditions of the soil on site. Calcium ion concentration in the medium was measured using a Horiba LAQUATwin calcium ion meter.

**Table 1:** Nutrient medium composition

Ingredient	Quantity (g/l)
Peptone	5.0
Sodium chloride	5.0
Beef extract	1.5
Yeast extract	1.5

### Material analysis

We used scanning electron microscopy (SEM) to inspect the microstructure of the biominerals formed. It was carried out using a ThermoScientific Pharos FEG-SEM with high vacuum mode and 15 kV acceleration voltage. We performed energy-dispersive x-ray spectroscopy (EDX) analysis using a Silicon drift detector (SDD) and 30-s integration time. Furthermore, the bioprecipitates were analysed by a Cary 630 FTIR spectrometer (Agilent Technologies) with range 600–4000 cm^−1^ and resolution of 4 cm^−1^. Raman spectroscopy was carried out to identify the bioprecipitates using a Horiba LabRAM ARAMIS confocal Raman microscope (50X objective, 633-nm laser − 1% power, 100-μm pinhole, 600 l/mm grating).

### Testing for soil biocementation

Specimens of 100-mm height and 50-mm diameter were compacted at a dry density of 1.34 g/cm^3^ (which was the dry density of the soil in the field) and at the natural gravimetric soil water content. A 2-stage injection was used to implement the treatments: first, the bacteria (cell concentration of 9 × 10^8^ cells/l) were injected (stage 1), followed by the cementation solution (in this instance, 0.1 M Ca(CH_3_COO)_2_ and NaHCO_3_ solutions) (stage 2). Solutions were injected by syringe into the top of the soil specimens. A two-stage injection was deemed preferable to allow bacteria to attach to the particles before cementing solution is sent through (thus creating nucleation sites for subsequent calcite precipitation) but also to prevent calcite formation in the syringe before injection in the soils as the CA-induced chemical reaction was observed to be very fast. Specimen curing lasted 14 days. As the untreated (control) specimens were not sufficiently cohesive to test in unconfined compressive strength testing apparatus and were crumbling as soon as compression was applied, the approximate unconfined compressive strength of all specimens (treated and untreated) was tested using a pocket penetrometer. After pocket penetrometer testing, samples across the specimen were collected and their oven-dried masses determined. They were then subjected to acid wash using a 2 M HCl solution, oven-dried and their mass measured again. The difference in these two oven-dried masses was identified as the CaCO_3_ mass produced by biocementation. Filter paper was used while rinsing repeatedly the dissolved CaCO_3_ and HCl solution to flush the dissolved salts out the soil without losing the soil particles.

## Results and discussion

### Microbiological study

#### Isolated strain identification—DNA sequencing results

**Fig. 3:**
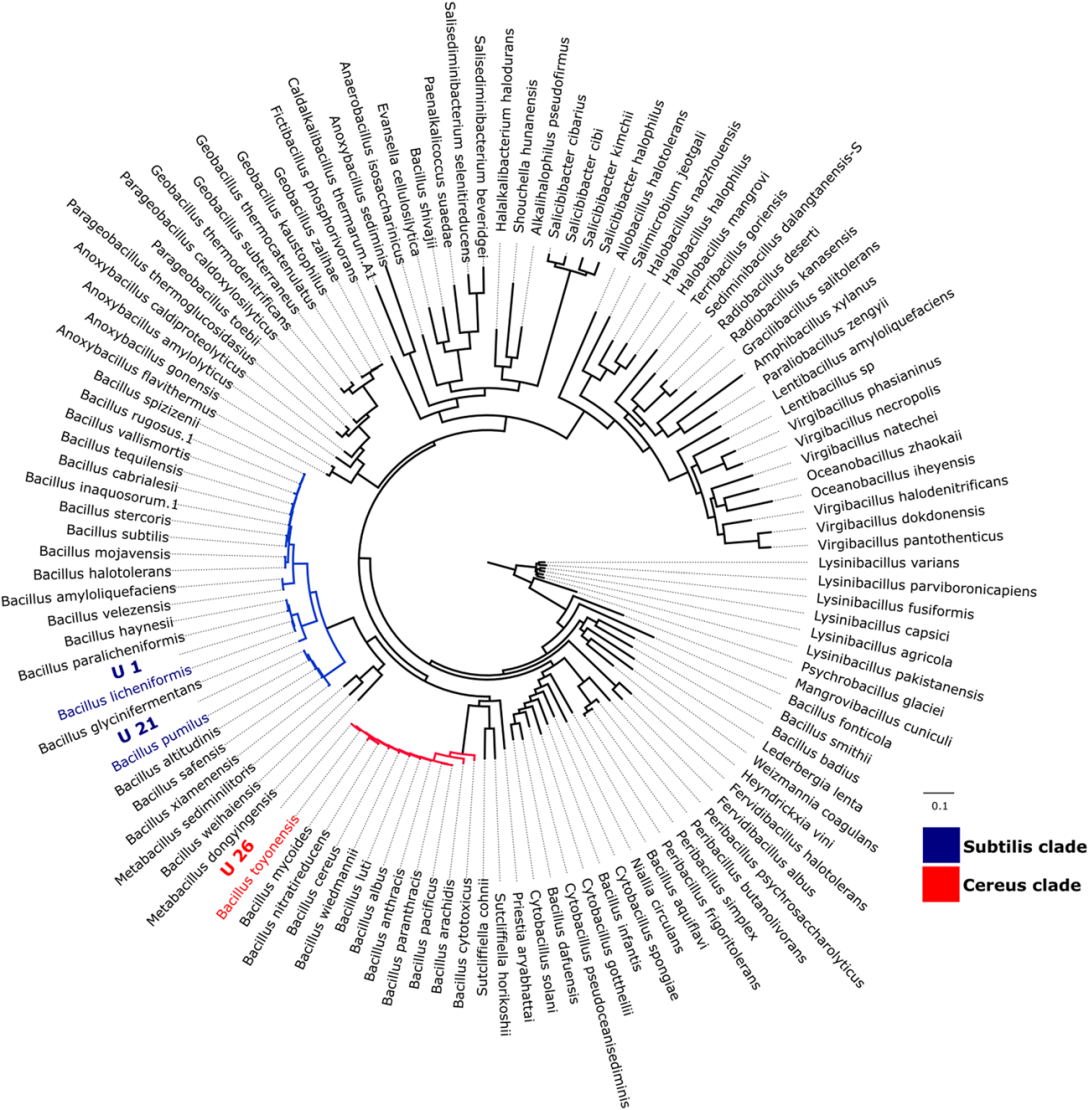
Results of maximum likelihood-based phylogenetic analysis of 119 Bacillaceae including the three selected isolates (U-1, U-21, U-26). The tree is based on 74 translated and concatenated protein coding gene sequences and was rooted on *Lysinibacillus*. No support values are shown; Phylogenetic tree with support values is provided as supplementary material (Online Resource 1b)

The 40 CA-producing bacteria were subjected to preliminary identification at the end of the incubation period, based on MALDI-TOF testing. All strains that returned MALDI-TOF matches of secure or probable genus identification were identified as of the *Bacillus* genus. The three selected strains were then identified by DNA sequencing. In total, 3,991,848 (for U-1), 3,417,220 (for U-21) and 4,663,946 (for U-26) read pairs were generated, and of these read pairs, 3,987,295 (99.89%), 3,360,137 (98.33%) and 4,606,773 (98.77%) passed the quality control step. The data was assembled and assembly quality assessed. The U-1 assembly consists of 86 contigs with total length of 4,171,408 bp, a N50 of 92,570, a GC% of 46%. Of the reads, 99.79% could be mapped back to the contigs, and the average coverage was 194. The U-21 assembly consists of 124 contigs with a total length of 3,877,410 bp, a N50 of 67,646, a GC% of 41%. Of the reads, 99.76% could be mapped back to the contigs, and the average coverage was 176. The U-26 assembly consists of 286 contigs with a total length of 6,217,871 bp, a N50 of 70,265, a GC% of 35%. Of the reads, 99.86% could be mapped back to the contigs, and the average coverage was 150. The genomes were annotated and completeness estimated using CheckM (U-1: 98.86%; U-21: 95.61%; U-26: 99.31%), which also revealed low contamination (U-1: 1.20%; U-21: 3.53%; U-26: 0.54%). Strain heterogeneity was estimated to be 5.26% for U-1, 3.70% for U-21 and 75% for U-26. The CheckM genome completeness estimates were corroborated by HMMER searches, which also indicated that the genomes are largely complete: Of the 74 genes included in the phylogenetic analysis, 73 were recovered for U-1 (with 0% redundancy) and U-21 (with 1.35% redundancy). All 74 genes were recovered for U-26 (with 5.41% redundancy). These values are within the range seen for the 116 genomes that were downloaded from the NCBI Datasets database (for genome summary, see supplementary material in Online Resource 1a). The final data matrix had a length of 12,444 amino acids and was used for phylogenetic tree reconstruction. The robust placement of the strains in the tree (Fig. 3) suggests that U-1 is *Bacillus licheniformis*, U-21 is *Bacillus pumilus* and U-26 is part of the *Bacillus cereus* group and most likely is *Bacillus toyonensis*. U-1 and U-21 have five and three CA, respectively. This is similar to the reference genomes in the NCBI databases (*Bacillus licheniformis*: GCF_002074095.1, *Bacillus pumilus*: GCF_003020795.1). U-26 has five CA. The *B. toyonensis* reference genome (GCF_016605985.1) in NCBI databases has only four CA annotated, but checking using blast the five CA are all found in other publicly available *B. toyonensis* strains. The protein sequences are shown in Table S1 of supplementary material (Online Resource 2).

#### Bacterial growth and CA enzymatic activity

**Fig. 4:**
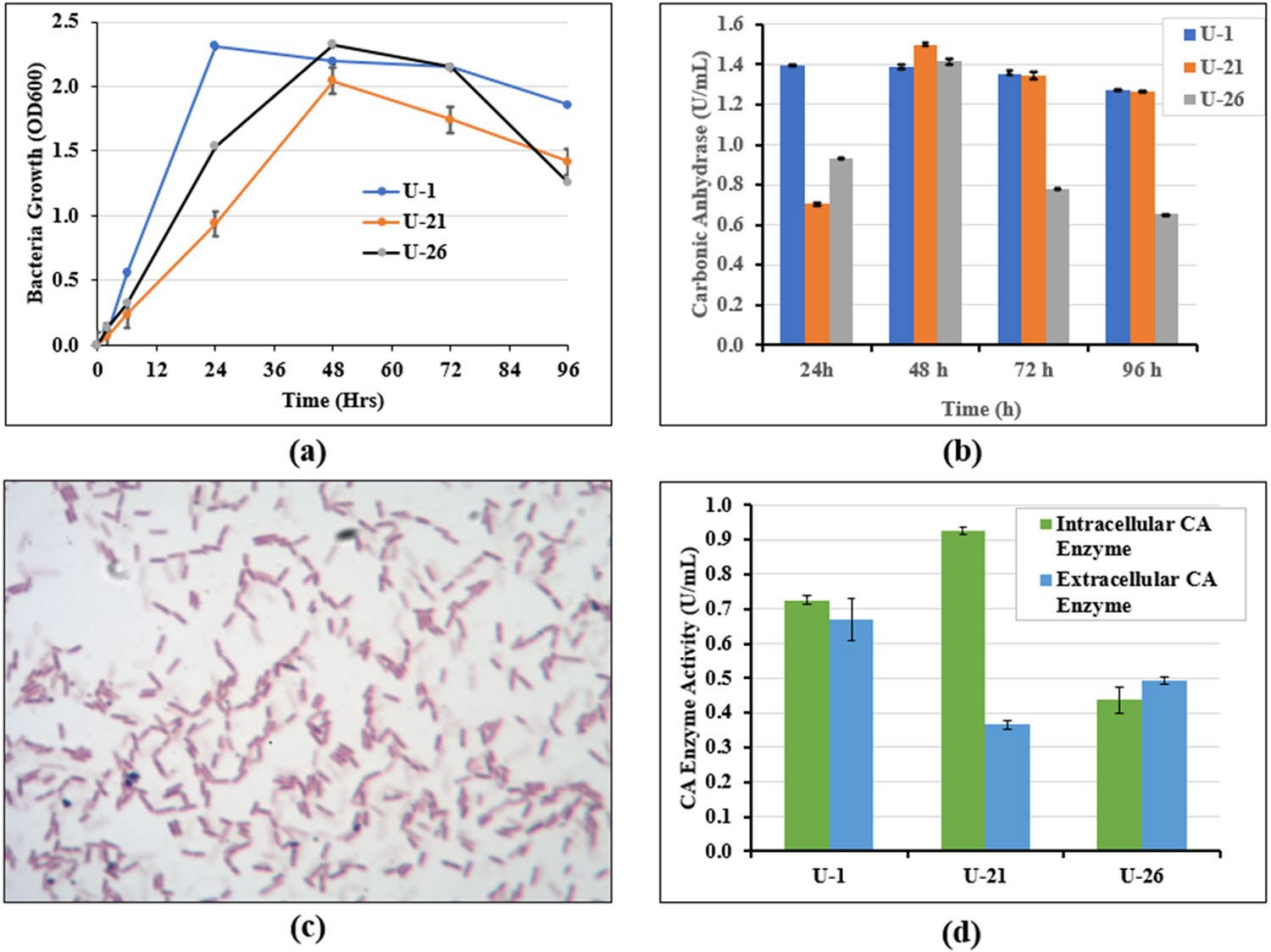
Bacterial growth and CA activity: **a** microbial growth curves; **b** variation of CA activity in time; **c** indicative gram-stained photo of *B. pumilus* isolate; **d** intracellular and extracellular CA activity

The results of microbial growth, CA enzyme activity and Gram stain of *B. licheniformis* (U-1), *B. pumilus* (U-21) and *B. toyonensis* (U-26) are summarised in Fig. 4 a–d. The microbial growth curves (Fig. 4a) show the typical lag, exponential, stationary and death phases. Maximum growth was observed at 24 h for *B. licheniformis* and at 48 h for *B. pumilus* and *B. toyonensis*.

Figure 4 b shows that *B. licheniformis*, *B. toyonensis* and *B. pumilus* had high specific carbonic anhydrase enzymatic activity (respectively 1.39, 1.42 and 1.50 U/ml) and can be used for biocementation. The CA activity of the isolated strains is higher than that reported for *B. schlegelii* isolated from garden soil (0.0453 U/ml) (Nathan and Ammini 2019) and *B. altitudinis* isolated from mangrove sediments (0.695 U/ml) (Muley et al 2014). However, the three isolates showed a lower CA activity compared to *Bacillus* sp. AP6 (5.61 U/ml) and *Bacillus mucilaginosus* used respectively for biocementation and CO_2_ capture (Achal and Pan 2011). In general, *Bacilli* species were found to be tolerant to high CO_2_ concentrations in previous investigations (see e.g. Hu et al 2020). The three isolates are thus good candidates for both carbon sequestration and biocementation applications.

The results of the intracellular and extracellular enzyme activities (Fig. 4d), which were determined to elucidate the mechanism by which the isolates secrete CA, show that consistently with Klier et al. (2014), the isolates can secrete both intracellular and extracellular CA and can thus be also used to produce crude CA for biocementation applications.

#### Optimization for CA production

**Fig. 5:**
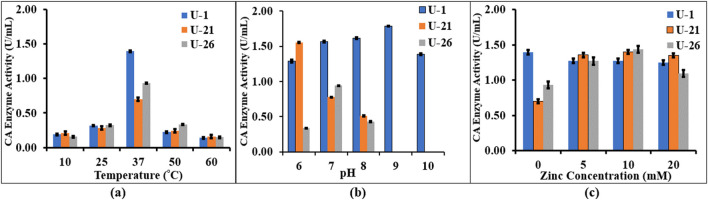
Impact of different factors on CA enzyme activity of the three isolates: **a** effect of temperature, **b** effect of pH, **c** effect of Zn as a cofactor on the three isolates. Values shown are mean ± SD (*n* = 3)

Three factors were optimised to determine the most optimal parameters for the growth of the selected strains, namely, pH, temperature, and Zn cofactor addition (Fig. 5a–d). Figure 5 a shows that all three isolates had lower CA activity at temperatures higher than 50 °C or lower than 25 °C; optimal microbial growth was observed at 37 °C. However, temperatures below 20 °C, which would be most common in the soils in situ, appeared to have little effect on the comparative activity of the different bacteria. Figure 5 b shows that all three bacteria could grow at pH 6, 7 and 8; however, only *B. licheniformis* could grow at pH 9–10, but no growth was observed for *B. licheniformis* at pH values > 11. The optimal pH for *B. licheniformis*, *B. pumilus* and *B. toyonensis* was determined as pH 9 (where CA activity increased to 1.79 U/ml), pH 6 and pH 7 respectively.

Figure 5 c shows the effect of supplementing Zn cofactor for improving CA production. As shown in the figure, there was an approximate 40% increase in CA production in *B. pumilus* and *B. toyonensis* when cofactor was added at increasing concentrations, optimally at a dosage of 10 mM Zn^2+^. A previous study (Jaya et al. 2019a, b) similarly reported that 10 mM Zn^2+^ improved the growth of *B. altitudinis* M8 strain isolated from the mangrove soil microcosm. The increased microbial growth and CA production upon Zn addition as a cofactor has been attributed to the fact that Zn ions are able to stabilise the structure of CA enzyme by resisting electrostatic repulsion (see e.g. Tu et al. 2012). Interestingly, however, no increase was shown in *B. licheniformis*; in fact, the results imply that Zn addition inhibited microbial growth. All subsequent investigations in this study used the optimized conditions for each strain in terms of pH, temperature and Zn cofactors based on this investigation.

**Fig. 6:**
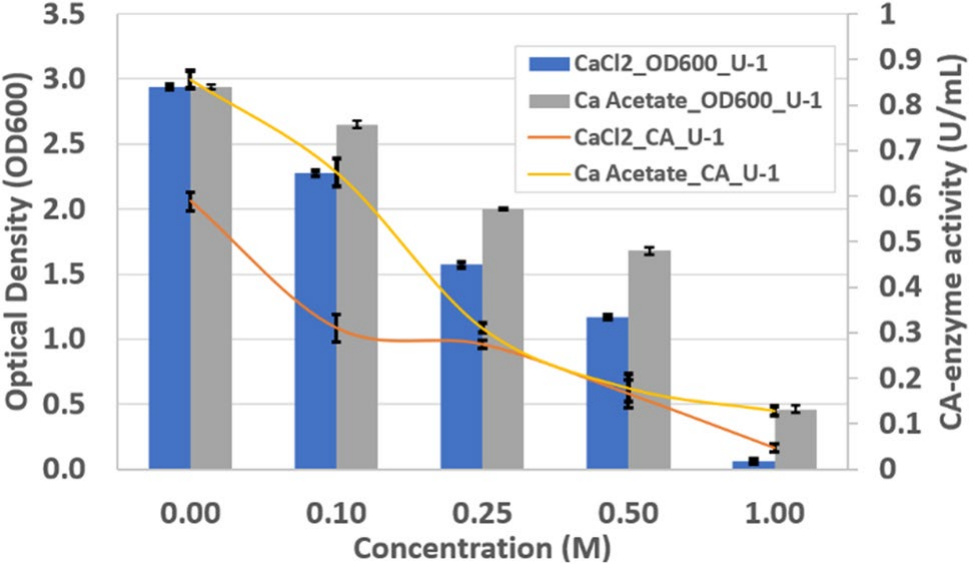
Impact of different calcium sources on the growth and CA activity of *B. licheniformis* (U-1)

Furthermore, Fig. 6 shows that calcium acetate was more favourable than calcium chloride in terms of bacterial growth and enzymatic activity for the solution concentrations tested, possibly as it acts as an additional source of carbon for the bacteria.

### CaCO_3_ bioprecipitation results

**Fig. 7:**
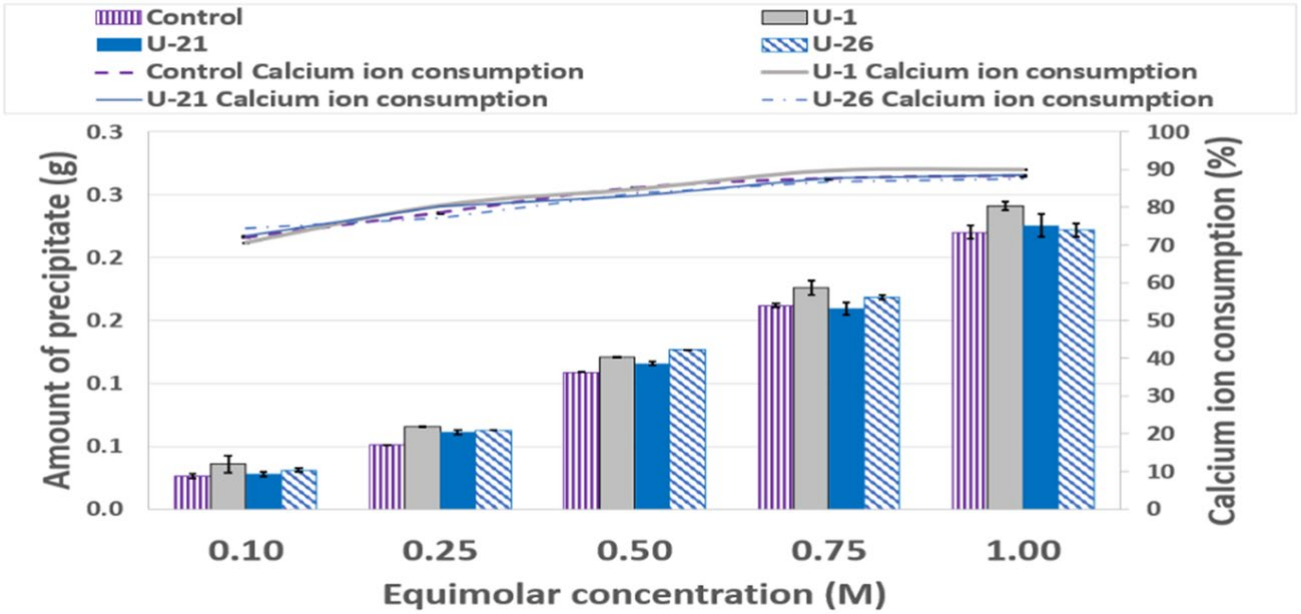
Deposited Ca.^2+^ at different cementation solution concentrations for *Bacillus licheniformis* (U-1), *Bacillus pumilus* (U-21), and *Bacillus toyonensis* (U-26)

CaCO_3_ bioprecipitation was studied to assess if the three isolated strains can potentially biocement soil. Figure 7 shows the quantity of deposited Ca^2+^ using different molarity cementation solutions for *Bacillus licheniformis*, *Bacillus pumilus* and *Bacillus toyonensis*, respectively. Deposited bioprecipitated Ca^2+^ increased as solution concentration increased in comparison to purely chemical reaction precipitate, in particular for *Bacillus licheniformis* although for the other two strains, the difference with comparison to the chemical reaction precipitates became smaller as solution concentrations increased. The higher deposited Ca^2+^ amount compared to the purely chemical reaction could be due to the fact that microbial cells become nucleation sites for the crystallisation of CaCO_3_ (Aloisi et al 2009). However, the marginal increase in deposited Ca^2+^ compared to the purely chemical reaction at higher molarities at least for *Bacillus pumilus* (U-21) and *Bacillus toyonensis* (U-26) could be due to enzymatic activity inhibition in solutions of high calcium ion concentrations, as reported in the literature (Whiffin 2004). Zheng and Qian (2020) explained that high Ca^2+^ concentrations increase osmotic pressure around the bacteria cell membrane, triggering flow from low to high Ca^2+^ concentration areas through the cell membrane. This causes cell dehydration and plasma wall separation; microbes may thus stop growing and die. Additional investigations of bioprecipitation by ureolytic bacteria isolated from the soil in 1 M cementing solutions showed that the CA route compared favourably against the ureolytic route in terms of precipitate amount (Online Resource 3).

**Fig. 8:**
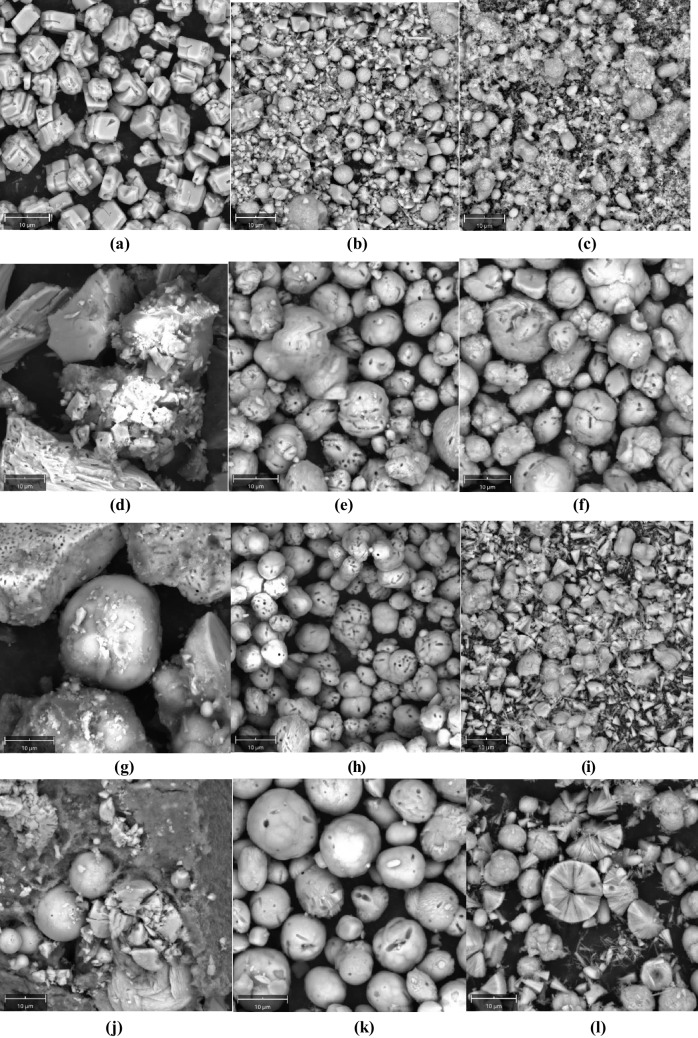
SEM results at different cementation solution molarities: **a**–**c** control, 0.1 M, 0.5 M, 1 M respectively; **d**–**f** *Bacillus licheniformis* (U-1), 0.1 M, 0.5 M, 1 M respectively; **g**–**i** *Bacillus pumilus* (U-21, 0.1 M, 0.5 M, 1 M respectively; **j**–**l** *Bacillus toyonensis* (U-26), 0.1 M, 0.5 M, 1 M respectively

**Fig. 9:**
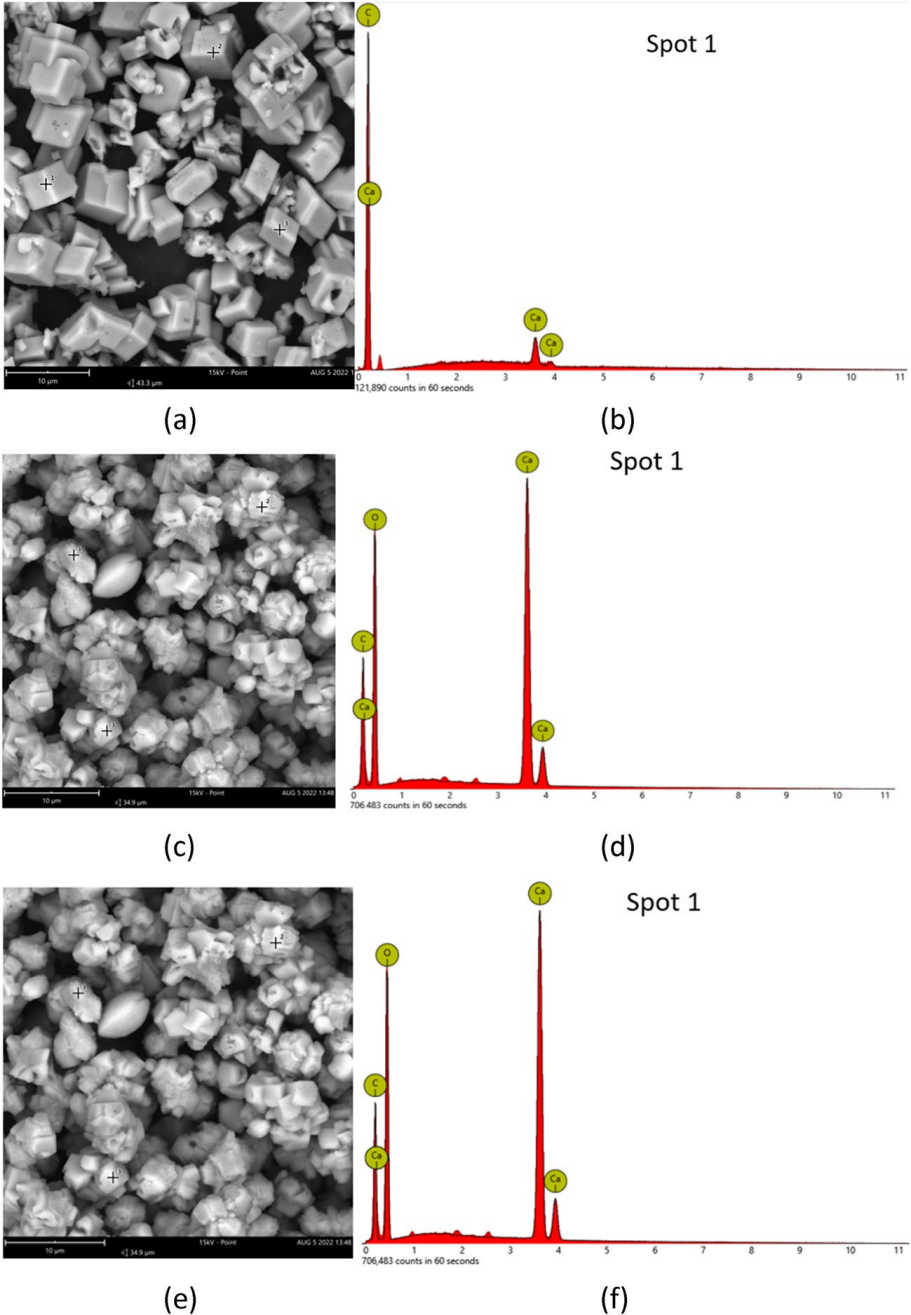
Indicative SEM–EDS results confirming CaCO_3_ precipitation: **a**, **b** control; **c**, **d** *Bacillus licheniformis* (U-1); **e**, **f** *Bacillus toyonensis* (U-26)

SEM was used to confirm the CaCO_3_ bioprecipitation at different cementing solution molarities; the results are shown in Fig. 8, which clearly demonstrate that cementation solution concentration affects the crystal morphology and size. The purely chemical reaction of sodium bicarbonate and calcium acetate (labelled as “control” analysis in Fig. 8a–c) produced mainly rhombohedral calcite crystals at low molarities; at higher molarities, some more spherical crystals and amorphous phases are also noted. Bacteria produced different bioprecipitates. For *Bacillus licheniformis* (U-1) (Fig. 8 d–f) at low molarities, rhombohedral crystals were also observed, but more spherical crystals were observed at higher molarities probably denoting the presence of vaterite, a more unstable CaCO_3_ phase. For *Bacillus pumilus* (U-21) (Fig. 8 g–i) and *Bacillus toyonensis* (U-26) (Fig. 8 j–l), the formation of calcite at low molarities is less well pronounced; at intermediate molarities, spherical precipitates are again observed whereas at 1.0 M concentration, crystal morphology was more irregular with various forms of crystals, including some needle-like crystals especially for *Bacillus toyonensis* (U-26) which are more consistent with the morphology of aragonite crystals. EDS analysis, with indicative results shown in Fig. 9, confirmed the CaCO_3_ formation (see elemental composition at indicative spots on the respective SEM samples).

**Fig. 10:**
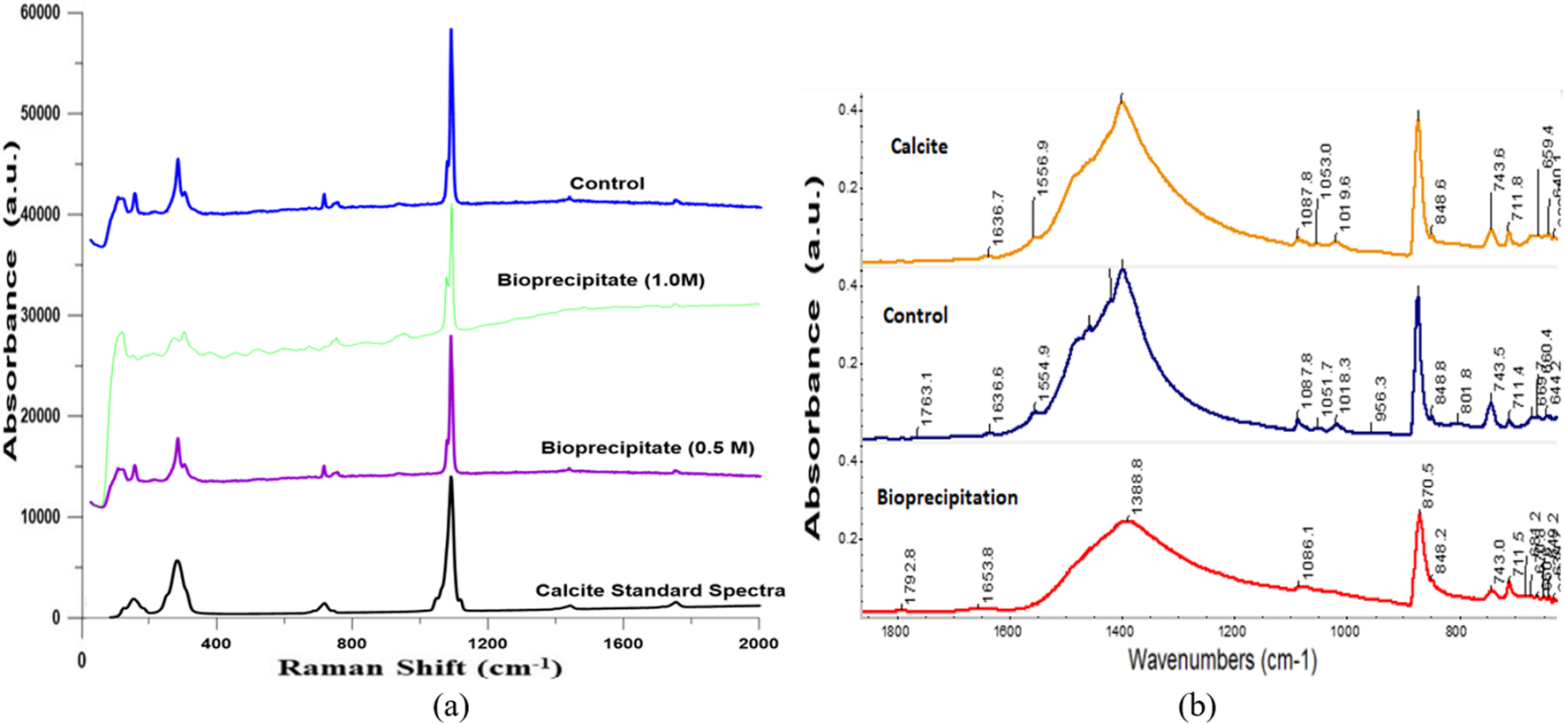
**a** Raman spectra of control, bioprecipitate and calcite standard spectra. **b** FTIR spectra of control, bioprecipitate and calcite standards spectra (a.u. stands for ‘arbitrary units’)

Raman analysis results in Fig. 10a also showed spectra dominated by carbonates/calcium carbonate (calcite)-like signals in particular the highest shift intensity at 1088 cm⁻^1^, which indicates that carbonate minerals are present. It can be noted that the spectra of typical bioprecipitates as well as the control (purely chemical precipitate formation) match typical calcite spectra, e.g. the primary peaks at 1088 cm⁻^1^ but also those at 280 cm^−1^ and 153 cm^−1^ and 715 cm^−1^, indicating that the bioprecipitates are calcite (see also similar results in other microbially induced carbonate precipitation studies, e.g. Bai et al 2017). The Raman spectra results demonstrate that calcite was formed using CA-producing bacteria from the selected isolate from the soil. FTIR analysis (Fig. 10(b)) further confirms carbonate formation, and in particular calcite; in particular, C–O stretching vibrations around 1400 cm^−1^ indicate CO_3_^−2^ presence in both control and bioprecipitates; single peaks at around 1400, 870, 713 and 745 cm^−1^ are all observed in the standard calcite spectra indicating that CaCO_3_ in the form of calcite has apparently formed.

**Fig. 11:**
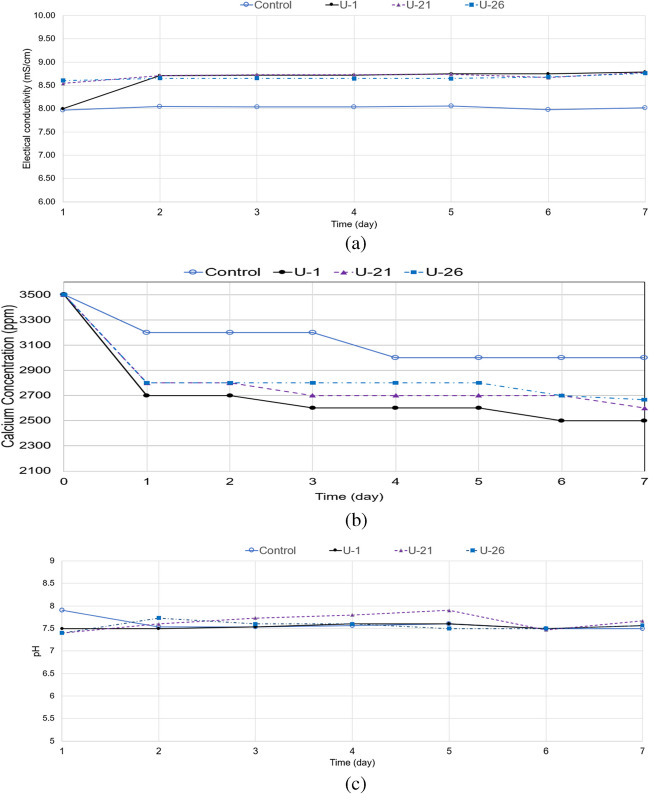
Bioprecipitation indicators due to atmospheric CO_2_ capture for the control mix (mostly chemical reaction—no bioaugmentation) and for bioaugmented samples, respectively, with *Bacillus licheniformis* (U-1), *Bacillus pumilus* (U-21) and *Bacillus toyonensis* (U-26): **a** electrical conductivity; **b** calcium concentration in the medium; **c** pH of the medium

The results of the bioprecipitation due to atmospheric CO_2_ capture (i.e. without the use of bicarbonate) are shown in Fig. 11. They report on the monitoring of bioprecipitation via electrical conductivity, pH and calcium concentration measurements in the medium in time. It is worth noting that in order to mimic site conditions, the bioaugmented substrates were exposed to the atmosphere, and sterility was not maintained. From Fig. 11, it can be seen that all bioaugmented samples manifest a change in their electric conductivity and calcium content indicating the formation of precipitation products; the sample which shows the most pronounced change in both electrical conductivity and calcium content for the first 6 days of exposure is that bioaugmented with *B. licheniformis*; the sample bioaugmented with *B. toyonensis* also mirrors closely the patterns of EC and calcium concentration change of the control mix (chemical reaction without bioaugmentation) although the change in EC is more pronounced compared to that of the control mix. On the other hand, the consumption of calcium ions does not happen at the same time as the EC change, and it also appears to be completed earlier than in the control mix.

### Soil biocementation

**Fig. 12:**
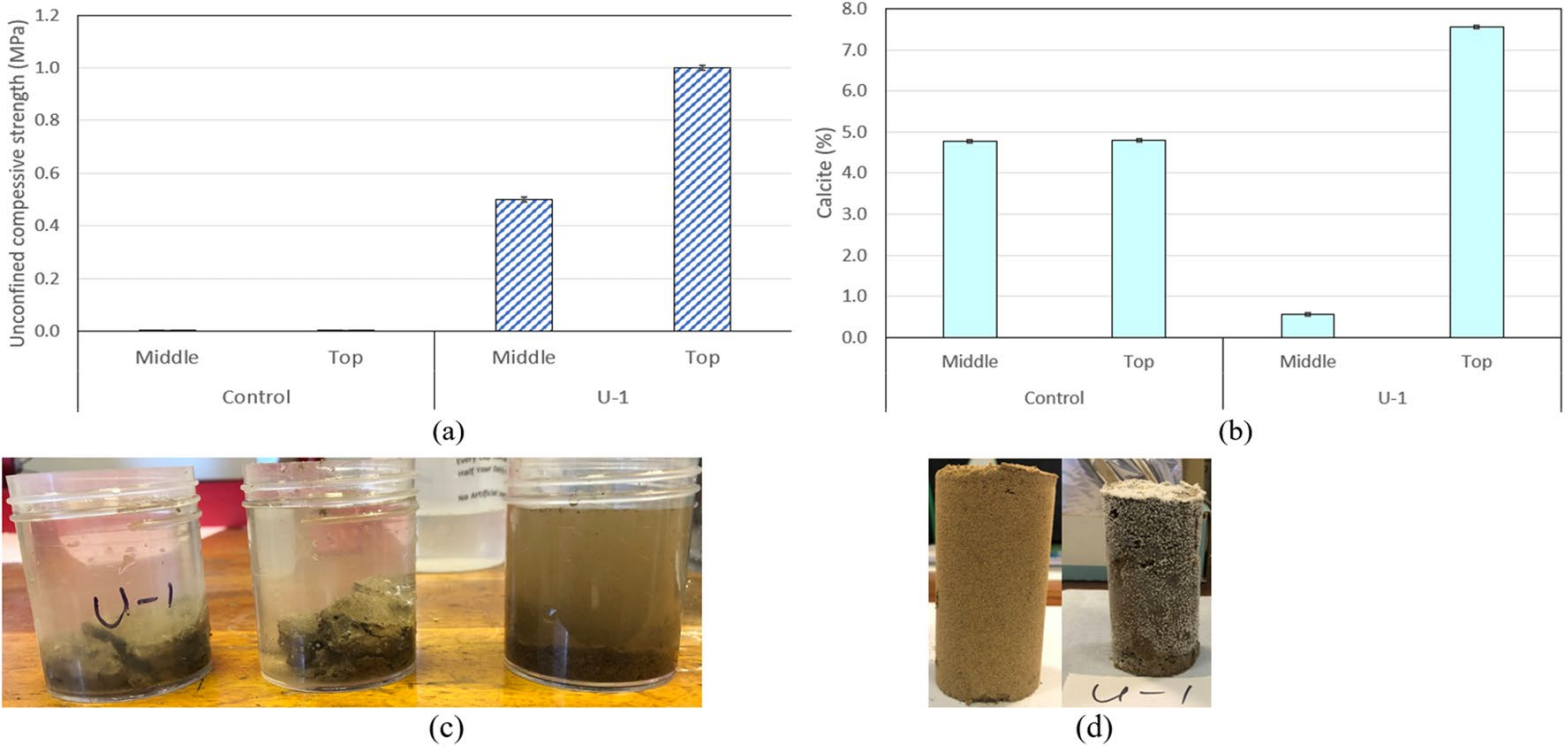
Biocementation study specimens: **a** pocket penetrometer results (UCS); **b** calcite content; **c** immersion test results of soil treated with *B. licheniformis*: samples from top (left), middle (middle) and bottom (right) of the soil specimen, respectively; **d** appearance of untreated (left) and treated (right) soil specimens

To prove soil biocementation, pocket penetrometer readings were taken at various parts of the injected specimens (Fig. 12a); the calcite content of the same specimens was then measured (Fig. 12b). From the figure, it can be seen that unconfined compressive strengths of 1 MPa and 0.5 MPa were obtained respectively at the top and middle of specimens treated with *B. licheniformis*. An increase of calcite content of 2.78% and 0.57% was noted respectively at the top and middle of specimens; this is consistent with crystals forming on the treated specimen surface (Fig. 12b), unlike the untreated soil specimen. However, the bottom of the treated specimens showed no evidence of biocementation, as no increase in calcite content was observed and pocket penetrometer testing was unfeasible (same as for the untreated soil specimens) because the bottom of the specimens lacked in cohesion and crumbled upon testing. Immersion of samples in water for 1 day proved that the strengths recorded for the top and middle of the specimens were due to cementation rather than suction effects, as the soil samples remained intact after immersion in water; conversely, the samples coming from the bottom of the specimens disintegrated in water, consistently with the lack of biocementation (see Fig. 12a). The non-uniformity of biocement distribution in soils is a known problem when injection is used for the implementation of the treatments; in this case, it was also exacerbated because of the fineness (thus, low hydraulic conductivity) of the soil. For this reason, we are developing instead electrokinetic implementation techniques (see Mwandira et al 2023a), which were shown to result in much improved treatment uniformity for fine-grained soils (Safdar et al 2021b). In the present work, however, the aim was the proof of soil biocementation using the autochthonous CA-producing strains, rather than the development of appropriate implementation techniques, which are the focus of future work. Based on the obtained results, biocementation was proven for the parts of the specimens that treatments have reached.

## Conclusion

This research work identified and studied carbonic anhydrase–producing bacteria isolated from a railway embankment foundation soil in East Anglia, towards future field biocementation application. The main focus of this paper was the study of the growth of the isolated autochthonous bacteria, the optimisation of carbonic anhydrase production and the study of bioprecipitates upon cementing solution implementation. Indicative results proving native soil biocementation using one of these strains were also presented.

The main findings of the paper were that:

- Most strains with CA activity isolated from the native soil were of the *Bacillus* genus; three of the most promising strains in terms of growth and carbonic anhydrase activity were identified as *Bacillus licheniformis*, *Bacillus pumilus* and *Bacillus toyonensis.*
- The study showed that the three specific *Bacillus* bacterial strains were able to produce CA for biocementation and capture CO_2_. Of these strains, *Bacillus licheniformis* had the added advantage of a more rapid growth (12 h) at a wider pH range, namely pH = 6–10, and did not require any Zn cofactor to enhance its activity.
- All three strains showed good intracellular as well as extracellular activity (an advantage if crude enzyme from bacteria was to be utilised) and had optimal CA activity at 37 °C.
- The results indicated that calcium acetate enhanced the growth of *Bacillus licheniformis* and its CA activity compared to calcium chloride for the range of molarities studied.
- Different bacterial strains and solution concentrations led to different bioprecipitate morphologies, but the presence of calcite was confirmed by material analysis.
- The bioaugmentation treatment with CA-producing bacteria considerably increased the UCS of the biocemented parts of the soil reaching up to 1 MPa of UCS and produced additional calcium carbonate of up to an extra 2.78% compared to that of the untreated soil. Immersion testing results concurred with soil biocementation.
- As CA homologues were present in the genome, these may be valuable for further functional analyses and studies to further understand the biocementation process with concurrent CO_2_ sequestration.

Overall, the results show promise that the use of CA-producing bacteria can produce biocements while capturing CO_2_ and that upon further development of soil treatment protocols and treatment implementation techniques, these biocements can be suitable to improve the engineering soil properties, including those of fine-grained soils. Achieving this would thus help decarbonise both the construction and maintenance of earthwork infrastructure, while making it more resilient to the effects of a changing climate.

### Supplementary Information

Below is the link to the electronic supplementary material.

Supplementary file1 (TSV 21 kb)

Supplementary file2 (PDF 10 kb)

Supplementary file3 (DOCX 15 kb)

Supplementary file4 (DOCX 94 kb)

## Data Availability

The sequencing data for the three isolated strains were deposited to NCBI SRA database (BioProject ID: PRJNA1034511). The assembled genome datasets were deposited to figSHare repository of Middlesex University (10.22023/mdx.24526309.v1). All data supporting the findings of this study is included in the paper. If any raw data files are required in a different format, these can be requested and can be made available by the first author upon reasonable request.
