# Supplementary figures and images for: A study of bacteria producing carbonic anhydrase enzyme for CaCO3 precipitation and soil biocementation
Source: Environ Sci Pollut Res Int. 2024 Jul 8;31(33):45818–33. doi: 10.1007/s11356-024-34077-0 (PMC11269399; doi:10.1007/s11356-024-34077-0)

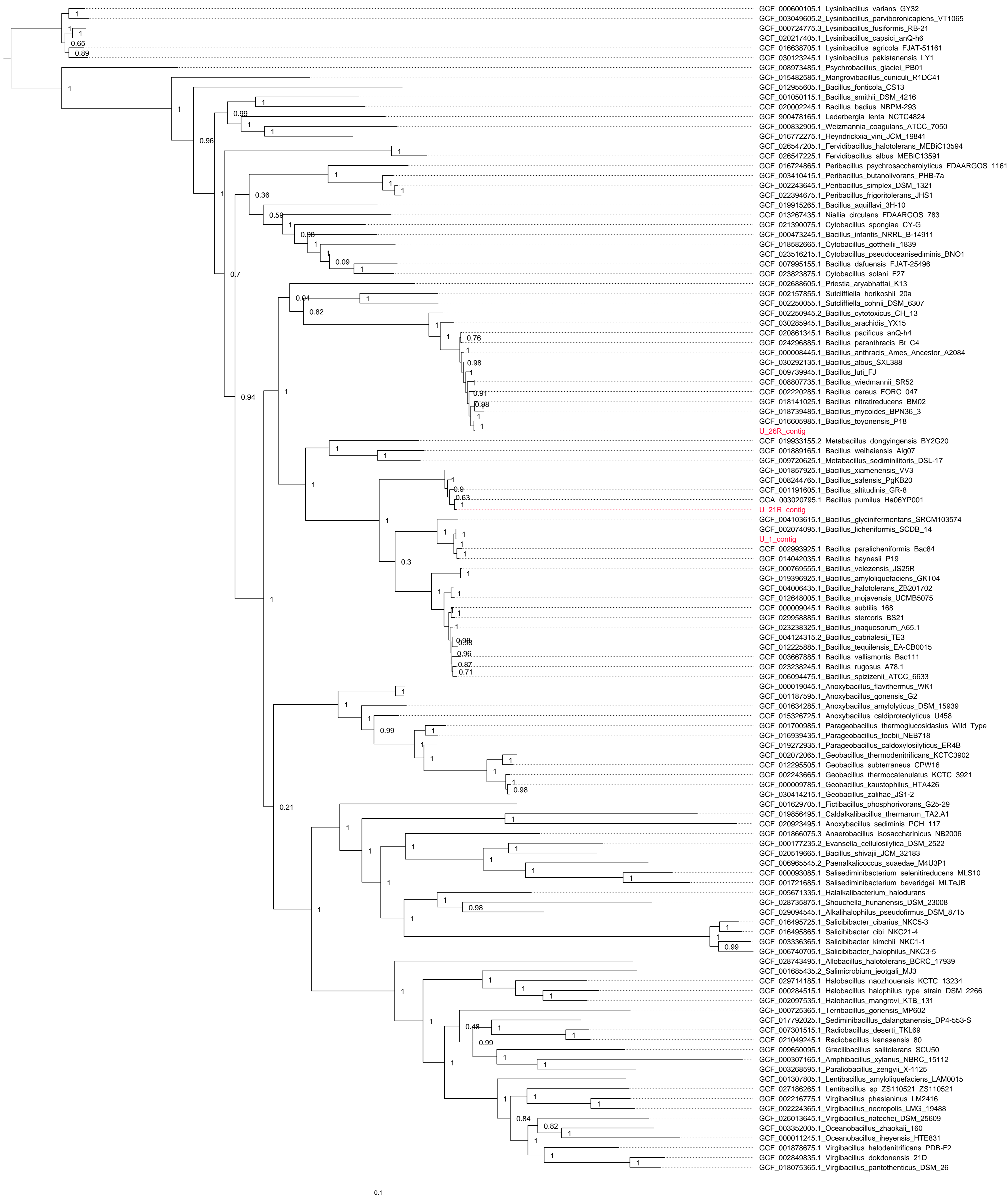

Supplement: Supplementary file 2 — Supplementary file2 (PDF 10 kb) [file 11356_2024_34077_MOESM2_ESM.pdf]
