# Supplementary material for: A study of bacteria producing carbonic anhydrase enzyme for CaCO3 precipitation and soil biocementation
Source: Environ Sci Pollut Res Int. 2024 Jul 8;31(33):45818–33. doi: 10.1007/s11356-024-34077-0 (PMC11269399; doi:10.1007/s11356-024-34077-0)
Supplement: Supplementary file 3 — Supplementary file3 (DOCX 15 kb) [file 11356_2024_34077_MOESM3_ESM.docx]

Table S1. Protein sequences for the CA in the three strains.

| **Strain** | **Protein sequences** |
| --- | --- |
| U-1 | >gnl\|extdb\|pgaptmp_000330 carbonic anhydrase [Bacillus licheniformis]  MAILTSILKHNQSFVEEKGYEFYETTKFPEKKLVILTCMDTRLLELLHHAMGLKNGDAKIIKNAGAVVSH  PFGSVMRSILVAVYELQAEEVCVIGHHECGMASLNASSILQKAKQRGVDDGCLELLQHSGIDLDTWMTGF  DSVEDSVSHSVNMIRKHPLLSSDVPVHGLVIDPKTGRLDVVEDGYSREDVSLSEVR  >gnl\|extdb\|pgaptmp_000657 carbonic anhydrase [Bacillus licheniformis]  MEENKKILMIIGMEKQLEQTICRAANIQPEDALVLKSVFPDISQPYGDFMRDIITLVHRNKAEEIWIVRD  KYEPSADEDISDLLNRDKDLKDRMQTLEYLFQNCSPEFTQGSIDEWLSPSENVAEGVQKSVAAIRRHPLI  PLHVKVKGFLIDKSVLTEVEALNKNGAR  >gnl\|extdb\|pgaptmp_001371 carbonic anhydrase [Bacillus licheniformis]  MKLSSKLILGLTVSSLAGKFLEKLLIQDNVSPNITASFNQEADIPDIDASSYIHHFASVIGSVVIGRNVF  IGPFSSIRGDVGLKIFISHDCNIQDGVVLHGLKNYEYNSPVTEHSVFKDRESYSIYIGEKVSLAPQCQIY  GPVRIDKNVFVGMQSLVFDAYIQEDTVIEPGAKIIGVTIPPKRFVSAGRVISNQEDANRLPEITDSYPYH  DLNSKMTSVNLELAKGYKKEERQWKL  >gnl\|extdb\|pgaptmp_003167 gamma carbonic anhydrase family protein [Bacillus licheniformis]  MIYPYKKTEPVIHETAFIADNAVITGDVTIGERSSIWFSSVIRGDVAPVRIGKGVNIQDLSCLHQSPERP  LVIEDGVTVGHQVTLHSSVIRKHALIGMGSIILDEAEIGEGAFIGAGSLVPPGKKIPSEHLAFGRPAKVI  RPLTDKDKQEMERIRKEYIEKGQYYKSLQKKD  >gnl\|extdb\|pgaptmp_003520 carbonic anhydrase [Bacillus licheniformis]  MKLLDNILQFNQQFVERQDYQKYQTSKFPDKRMVILSCMDTRLVELLPHAMNMRNGDVKIVKSAGALVAH  PFGSIMRSILVAVYELNANEVCVIGHYDCGMSKLSCDSFLDKVVKRGIPKERIETLEYSGVDFEQWLKSF  DSVEDSVRDSVSVIRNHPLMPEEVPVHGLVIDPETGRLDLIVNGYPEER |
| U-21 | >gnl\|extdb\|pgaptmp_000298 carbonic anhydrase [Bacillus pumilus]  MGSKLEQILQHNSKFVQERHYEPYKVGKFPEKKLVILTCMDTRLLELLPQSMGLRNGDAKIIKNAGAIVT  HPFGSVMRSILLAIYELKAEEVCIVGHHECGMAGLAADPLLEKAKARGIEEKCLSIVKNSGVDLKGWLTG  FDSVEESVSQSVKLVKEHPLMPSDVAVHGLVIHPATGKLDVVVKDNQIDAQYT  >gnl\|extdb\|pgaptmp_004059 gamma carbonic anhydrase family protein [Bacillus pumilus]  MIYPYHQFTPEIHESVFVADNATITGDVTIGEYSSVWFQTVIRGDVAPVRIGKNVNIQDLSCLHQSPGKT  LLIEDGATIGHQVTLHSSIIRKNALIGMGSIILDGAEIGEGAFIGAGSLVPQGKVIPKGSLAFGRPAKVV  RQLTDEDIQDMDRIRREYVEKGQYYRSLISR  >gnl\|extdb\|pgaptmp_004083 carbonic anhydrase [Bacillus pumilus]  MKLLEEIIEYNQQFIEEKKYEEFTTTKFPQKKAVVLSCMDTRLVELLPRAMNMKNGDIKIVKSAGALVSH  PFGSIMRSILVAVYELNADEVYVIGHHDCGMSKIDSQTLLNKAIERGIPEKRIEVLEYSGIDFKQWLKSF  SSVEESVKDSVSVVKNHPLLPSNVPVHGLVIDPGTGKLDVVVNGYEEK |
| U-26 | >gnl\|extdb\|pgaptmp_000008 gamma carbonic anhydrase family protein [Bacillus toyonensis]  MIYPYKEKNPKISSSAFIADYVTITGDVSIGEEASIWFNTVIRGDVSPTIIGDRVNVQDQCTLHQSPQYP  LILEDDVTVGHQVILHSCHIKKDALIGMGSIILDGAEIGEGAFIGAGSLVSQGKKIPPNTLAFGRPAKVI  RELTEEDRKDMERIRTQYVEKGQYYKSLQK  >gnl\|extdb\|pgaptmp_002870 carbonic anhydrase [Bacillus toyonensis]  MLLQEILSFNEQFVENKEYAPFEATKMPKKRMVVVSCMDARLIELLPKALDIHDGDAKVIRNAGGKIASP  FDSVMQSVIASVYDLNADEIFLIGHHKCGASQTNPKGTIQKILDRGVASSEILSAIEYAGVDLEKWLFGF  DDVCDSTQANVDLVRNHPLIPKDVPVHGLVIDPHTGKLDLVVDGYKTLNGMKN  >gnl\|extdb\|pgaptmp_003542 carbonic anhydrase [Bacillus toyonensis]  MKSLEEILQYNEKFVEEKKYEEYETGKFPNKKMVIISCMDTRLVELLPKAMNMRNGDVKIIKVAGAVISH  PFGSIMRSILVAVYELGADEVCVVGHHDCGMAKIQASSTIEKMKERGITNEKLDTLRYSGIDLERFLQGF  SSVEESVEHSVSILRNHPLLPEEVPVHGLVIDPDTGKLDLVVNGYDN  >gnl\|extdb\|pgaptmp_004448 carbonic anhydrase [Bacillus toyonensis]  MNSKNKKVLLLTDIEHGIEPIIQQVTNSQQENMLTIHSYDSVIVHPYGDIMRSVIIAIYQENVEEIFVVG  IEDKETSSVNLQTQHDFIKDNIKLDYLFKNCMPEFSSGSLNAWLSGQENVSENIKKSIDMIRQHPLVPSD  IKVHGFMIDRTGGKETVAKVSANKVVEYI  >gnl\|extdb\|pgaptmp_006389 carbonic anhydrase [Bacillus toyonensis]  MPKMGNTFLTIQELEEKKEYLLGLSSVIPTWNTSYQFLFKEIQQELLSKVNEKIERNQFILNICADQQVG  A |
