# Supplementary material for: A study of bacteria producing carbonic anhydrase enzyme for CaCO3 precipitation and soil biocementation
Source: Environ Sci Pollut Res Int. 2024 Jul 8;31(33):45818–33. doi: 10.1007/s11356-024-34077-0 (PMC11269399; doi:10.1007/s11356-024-34077-0)
Supplement: Supplementary file 4 — Supplementary file4 (DOCX 94 kb) [file 11356_2024_34077_MOESM4_ESM.docx]

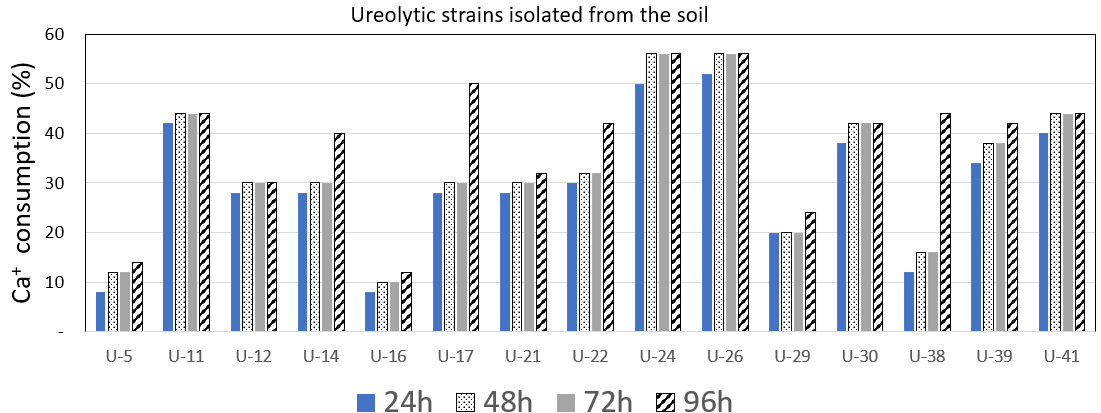


(a)


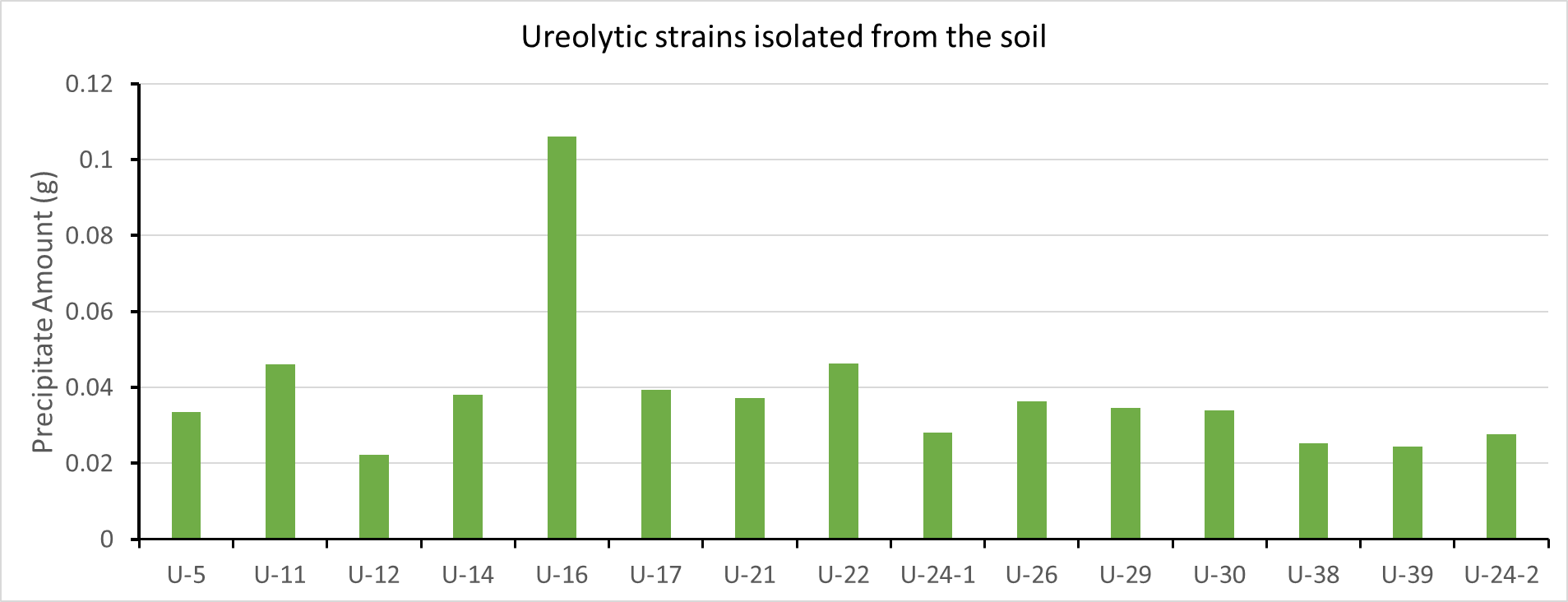


(b)

Figure S3. Study of ureolytic strains isolated from the soil: (a)Calcium ion consumption in time; (b) Precipitate amount.
